# Supplementary material for: Pinacolone-Alcohol Gas-Phase Solvation Balances as Experimental Dispersion Benchmarks
Source: Molecules. 2020 Nov 3;25(21):5095. doi: 10.3390/molecules25215095 (PMC7662480; doi:10.3390/molecules25215095)
Supplement: Supplementary file 1 [file molecules-25-05095-s001.pdf]

Supporting Information:

Pinacolone-alcohol gas-phase solvation balances as experimental dispersion benchmarks

C. Zimmermann, T. L. Fischer, M. A. Suhm*

E-mail: msuhm@gwdg.de

Table S1: ORCA 4.2.1[S1] and Turbomole[S2,S3] keywords used for the different electronic structure calculations, optimizations, transition state searches (TS) and relaxed scans (RS).

Level of approximation	Employed keywords
B3LYP-D3(BJ,ABC)/def2-TZVP (ORCA)	B3LYP D3BJ def2-TZVP abc grid5 NoFinalGrid UseSym VERYTIGHTSCF TIGHTOPT FREQ
B3LYP-D3(BJ,ABC)/def2-QZVP (ORCA)	B3LYP D3BJ def2-QZVPP abc grid5 NoFinalGrid UseSym VERYTIGHTSCF TIGHTOPT FREQ
DLPNO-CCSD(T) (ORCA)	DLPNO-CCSD(T) TightPNO aug-cc-pVQZ aug-cc-pVQZ/C TightSCF LED
TPSS-D3(BJ,ABC)/def2-TZVP (ORCA)	TPSS D3BJ def2-TZVP abc grid5 NoFinalGrid UseSym VERYTIGHTSCF TIGHTOPT NumFreq
TPSS-D3(BJ,ABC)/def2-QZVP (ORCA)	TPSS D3BJ def2-QZVPP abc grid5 NoFinalGrid UseSym VERYTIGHTSCF TIGHTOPT NumFreq
B97-3c-D3(BJ,ABC)/def2-mTZVP (TURBOMOLE)^{TS}	b973c def2-mTZVP grid m5 disp3 bj abc ri itrvec 1
B3LYP-D3(BJ,ABC)/def2-TZVP (ORCA)^{TS}	B3LYP D3BJ def2-TZVP abc grid5 NoFinalGrid UseSym OptTS VERYTIGHTSCF TIGHTOPT FREQ
B3LYP-D3(BJ,ABC)/def2-TZVP (TURBOMOLE)^{RS}	b3-lyp def2-mTZVP grid m5 disp3 bj abc ri

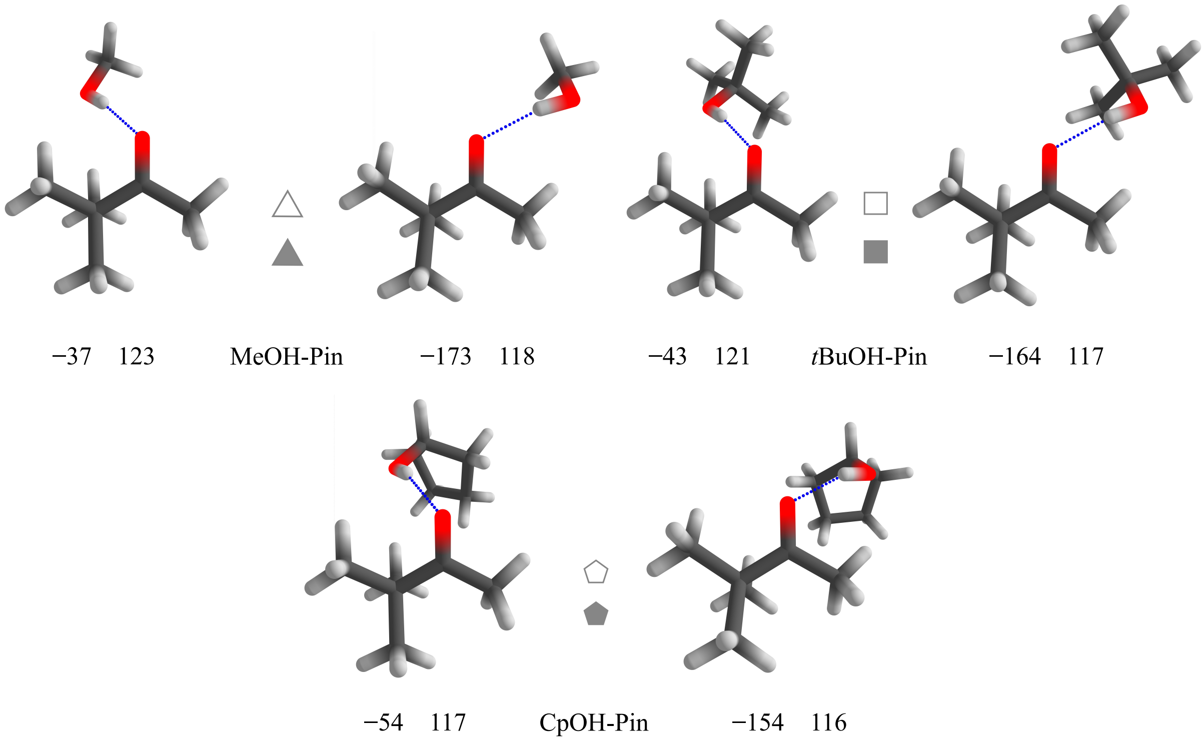

Fig. S1: Structures of the most stable alcohol-pinacolone dimers at B3LYP-D3(BJ,abc)/def2-TZVP level with the symbols used for them in the main text. Also given is the torsional angle τ of the hydrogen-bonded H relative to the *tert*-butyl group around the C=O group (C-C=O$\cdots$H, left value; the uniformly negative sign indicates that the solvating alcohol is pointing away from the reader) and the hydrogen bond angle α (C=O$\cdots$H, right value), both in $^\circ$.

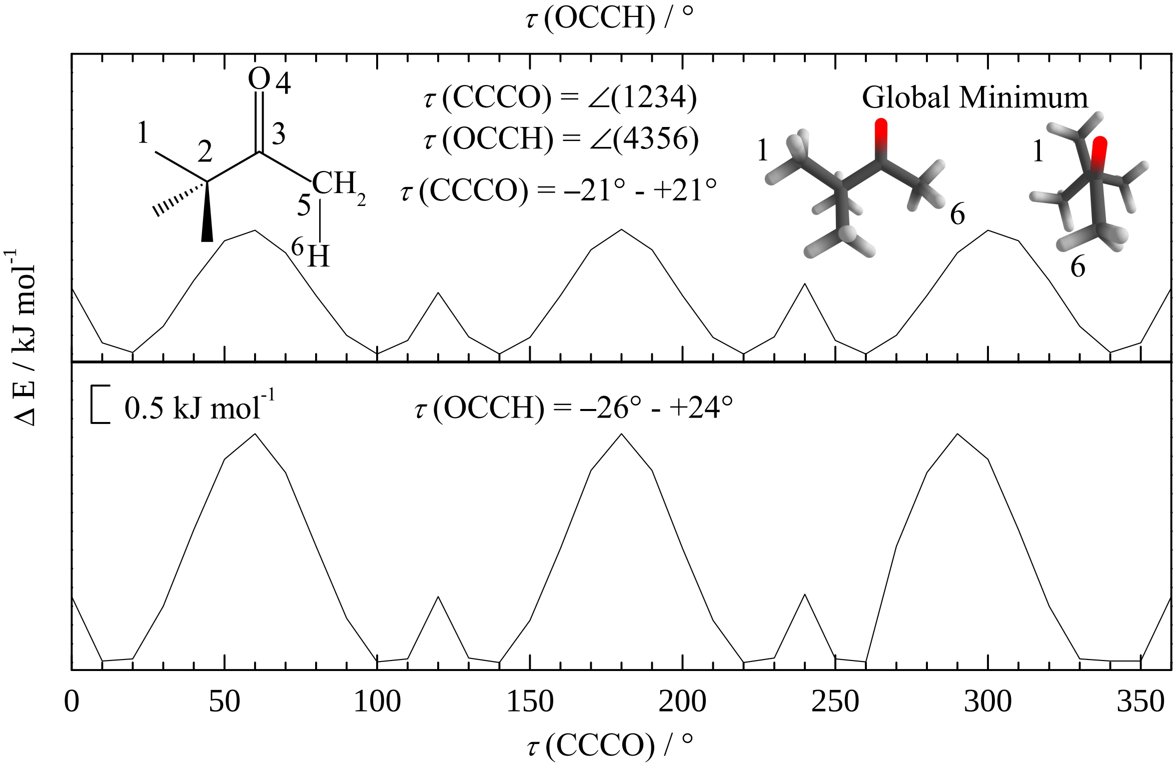

Fig. S2: Relaxed B3LYP-D3(BJ,ABC)/def2-TZVP scans along the OCCH (upper panel) and CCCO (lower panel) torsional angle for pinacolone monomer (with symmetry-breaking minima near $20°$, $100°$, $140°$, $220°$, $260°$). In a jet expansion, barriers below $4\,\mathrm{kJ\,mol^{-1}}$ can be largely overcome. This suggests that also in complexes with alcohols, the pinacolone isomerism can be relaxed efficiently and leads to no additional spectral complexity.

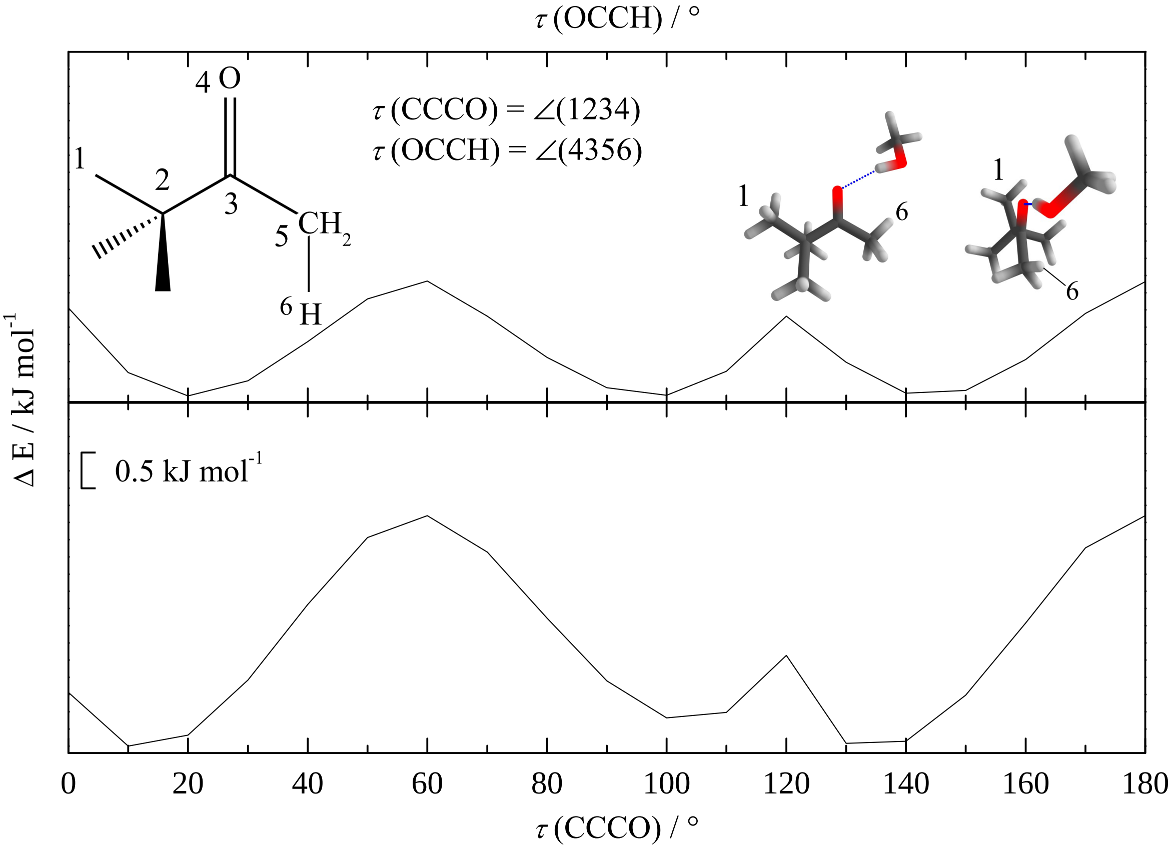

Fig. S3: Relaxed B3LYP-D3(BJ,ABC)/def2-TZVP scans along the OCCH (upper panel) and CCCO (lower panel) torsional angle for the Me-sided MeOH-pinacolone dimer structures. The persistently low barriers confirm that in complexes with alcohols, the pinacolone isomerism can be relaxed efficiently and likely leads to no additional spectral complexity.

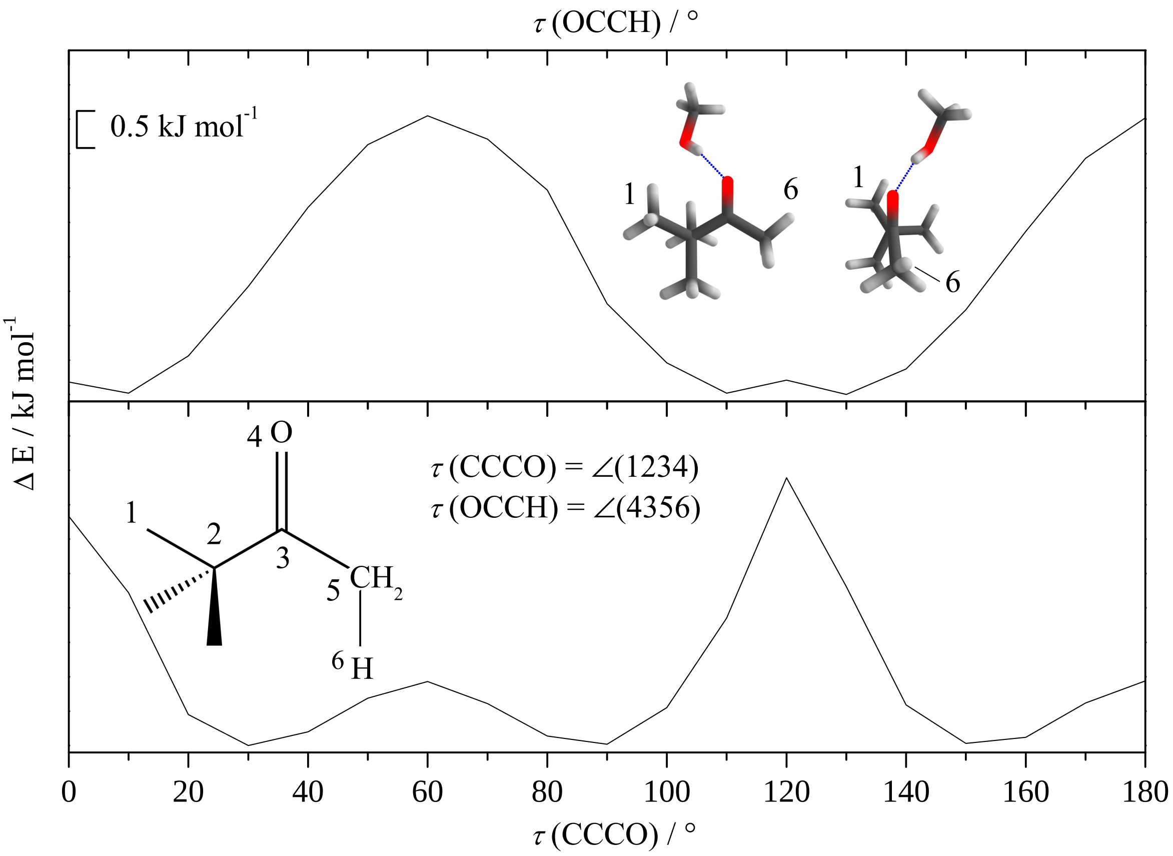

Fig. S4: Relaxed B3LYP-D3(BJ,ABC)/def2-TZVP scans along the OCCH (upper panel) and CCCO (lower panel) torsional angle for the *t*Bu-sided MeOH-pinacolone dimer structure, further confirming the insignificance of pinacolone isomerism for this study.

Table S2: Values for the hydrogen bond angle α between the ketone group and the hydrogen bonded H, and the associated H/*t*Bu torsional angle τ around the ketone group in °. The negative sign for τ corresponds to Fig. S1, where the alcohol substituent is pointing away from the reader. With increasing alcohol size, the torsional angle deviates further from planarity. For comparison, the angles in the CpOH complex with acetone (Me docking only) are α= 117° and τ= −177° at B3LYP/def2-TZVP level, so closer to the MeOH case.

			*t*Bu		Me	
Donor	Method	basis set	τ	α	τ	α
	B3LYP-D3(BJ)	def2-TZVP	−37	123	−173	118
MeOH	B3LYP-D3(BJ)	def2-QZVP	−37	124	−175	117
	TPSS-D3(BJ)	def2-TZVP	−37	123	−172	116
	TPSS-D3(BJ)	def2-QZVP	−35	125	−172	117
	B3LYP-D3(BJ)	def2-TZVP	−43	121	−164	117
*t*BuOH	B3LYP-D3(BJ)	def2-QZVP	−44	120	−163	117
	TPSS-D3(BJ)	def2-TZVP	−45	120	−163	116
	TPSS-D3(BJ)	def2-QZVP	−45	120	−163	116
	B3LYP-D3(BJ)	def2-TZVP	−54	117	−154	116
CpOH	B3LYP-D3(BJ)	def2-QZVP	−55	116	−153	116
	TPSS-D3(BJ)	def2-TZVP	−51	117	−154	115
	TPSS-D3(BJ)	def2-QZVP	−55	117	−153	115

Table S3: Methyl side docking preference in MeOH, *t*BuOH, CpOH-pinacolone complexes predicted by four DFT levels in $\mathrm{kJ\,mol^{-1}}$ relative to the *tert*-butyl side. ΔE^0 includes the harmonically approximated zero-point energy and ΔE^{el} excludes it. Negative values indicate a higher stability of the complex where the solvent docks on the methyl side of pinacolone. Zero-point energy corrections are typically small and do not change the isomer energy sequence. TPSS has a uniformly stronger methyl side docking preference than B3LYP.

Donor	Method	def2-TZVP		def2-QZVP	
		$\Delta E^{\mathrm{el}}_{\mathrm{Me}-t\mathrm{Bu}}$	$\Delta E^{0}_{\mathrm{Me}-t\mathrm{Bu}}$	$\Delta E^{\mathrm{el}}_{\mathrm{Me}-t\mathrm{Bu}}$	$\Delta E^{0}_{\mathrm{Me}-t\mathrm{Bu}}$
	B3LYP-D3(BJ)	−1.39	−1.40	−1.58	−1.59
MeOH	TPSS-D3(BJ)	−2.27	−2.04	−2.22	−2.16
	B3LYP-D3(BJ)	−0.66	−0.62	−0.80	−0.75
*t*BuOH	TPSS-D3(BJ)	−1.50	−1.32	−1.66	−1.51
	B3LYP-D3(BJ)	−0.35	−0.29	−0.38	−0.37
CpOH	TPSS-D3(BJ)	−1.37	−1.12	−1.48	−1.21

Table S4: Docking effect on the harmonic OH stretching wavenumbers ω and resulting wavenumber shifts $\Delta\omega$ for methyl (Me) and *tert*-butyl (*t*Bu) docking variants of alcohol-pinacolone complexes in $\mathrm{cm^{-1}}$. Negative signs indicate a higher wavenumber for *tert*-butyl docking.

Donor	Method	def2-TZVP			def2-QZVP		
		ω		$\Delta\omega_{\mathrm{Me}-t\mathrm{Bu}}$	ω		$\Delta\omega_{\mathrm{Me}-t\mathrm{Bu}}$
		Me	*t*Bu		Me	*t*Bu	
	B3LYP-D3(BJ)	3616	3645	−30	3627	3665	−37
MeOH	TPSS-D3(BJ)	3472	3514	−41	3482	3518	−37
	B3LYP-D3(BJ)	3617	3652	−35	3635	3673	−38
*t*BuOH	TPSS-D3(BJ)	3469	3516	−48	3478	3525	−47
	B3LYP-D3(BJ)	3643	3695	−51	3663	3714	−51
CpOH	TPSS-D3(BJ)	3497	3548	−50	3507	3570	−63

Table S5: Methyl side coordination preference for different explored alcohol-pinacolone complexes predicted at B3LYP-D3(BJ,abc)/def2-TZVP level relative to the *tert*-butyl side. Only alcohol conformers in an energy range of $< 2\,\mathrm{kJ\,mol^{-1}}$ are expected to be observed in experiment, thus only those conformers were considered in the search. ΔE^0 includes the harmonically approximated zero-point energy and ΔE^{el} excludes it. Zero-point energy corrections are typically small and do not change the energy sequence of the docking isomers. The preferred coordination is located on the methyl side of the pinacolone molecule for all presented systems, as shown by the uniformly negative sign. Also shown are harmonically predicted wavenumber differences $\Delta\omega_{\mathrm{Me}-t\mathrm{Bu}}$ between the docking sides, again uniformly negative. This shows that Me docking is always further downshifted in agreement with the higher stability. The shift is always predicted large enough to be experimentally resolved.

Donor	Cas No.	$\Delta E^{\mathrm{el}}_{\mathrm{Me}-t\mathrm{Bu}}$	$\Delta E^{0}_{\mathrm{Me}-t\mathrm{Bu}}$	$\Delta\omega_{\mathrm{Me}-t\mathrm{Bu}}$
Nonafluoro-*tert*-butyl alcohol	2378-02-1	−3.11	−3.38	−134
1,1,1,3,3,3-Hexafluoro-2-propanol (trans)	920-66-1	−3.38	−3.41	−111
1,1,1,3,3,3-Hexafluoro-2-propanol (gauche)	920-66-1	−1.86	−1.87	−112
Cyclopropanol (gauche)	16545-68-9	−2.09	−1.78	−14
(-)endo-Norborneol	61277-90-5	−2.00	−1.75	−68
Prop-2-in-1-ol	107-19-7	−1.59	−1.67	−72
1-Methylcyclopentanol	1462-03-9	−2.23	−1.63	−60
2,2,2-Trifluoroethanol	75-89-8	−1.95	−1.62	−108
Benzyl alcohol	100-51-6	−1.95	−1.54	−81
2-Methylcyclopentanol	24070-77-7	−1.93	−1.53	−21
2,5-Dimethylcyclopentanol (gauche)	63057-29-4	−1.58	−1.47	−35
(S)-(-)-1-Phenylethanol	1445-91-6	−1.23	−1.28	−55
Phenol	108-95-2	−0.98	−1.23	−62
Cyclopropanemethanol	2516-33-8	−1.44	−1.19	−48
1-Adamantanol	768-95-6	−1.00	−0.89	−28
Isopropyl alcohol (trans)	67-63-0	−0.87	−0.81	−38
Isopropyl alcohol (gauche)	67-63-0	−0.56	−0.50	−38
2-Adamantanol (gauche)	700-57-2	−0.20	−0.49	−23
2,5-Dimethylcyclopentanol (trans)	63057-29-4	−0.37	−0.26	−65

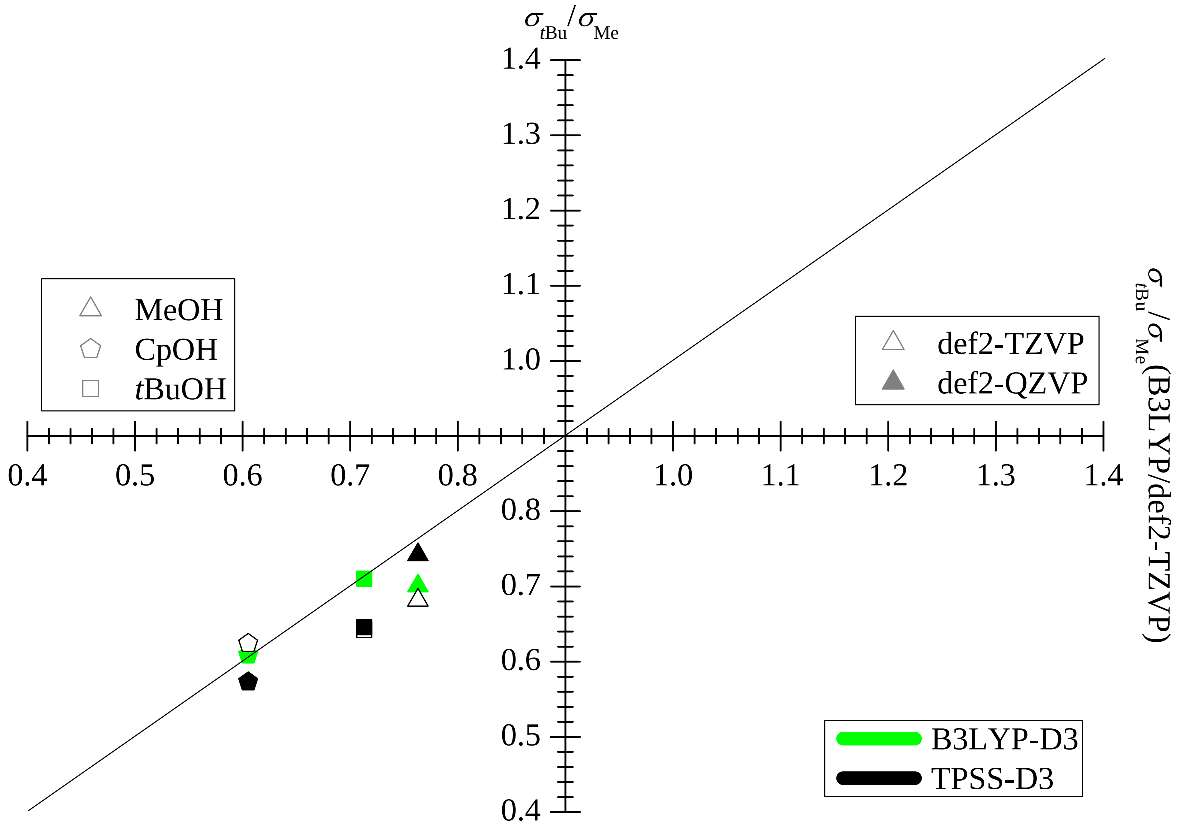

Fig. S5: B3LYP and TPSS infrared absorption cross section ratios $\sigma_{t\mathrm{Bu}}/\sigma_{\mathrm{Me}}$ with two different basis sets plotted against the B3LYP/def2-TZVP infrared absorption cross section ratios. The fairly good correlation along the bisector indicates that theoretical scatter in this quantity is small and the empty first quadrant shows that Me side docking always gives rise to stronger IR signals than *t*Bu side docking.

Table S6: Experimental OH stretching wavenumbers $\tilde{\nu}$ and resulting wavenumber shifts $\Delta\tilde{\nu}_{\mathrm{h-l}}$ for high (h) and low (l) lying spectral signals for alcohol-pinacolone 1:1 complexes in cm^{-1}. These shifts are essential to judge the predictive performance of the different DFT levels.

	$\tilde{\nu}$		$\Delta\tilde{\nu}_{\mathrm{h-l}}$
Donor	high	low	
MeOH	3561	3522	38
*t*BuOH	3537	3505	32
CpOH	3567	3527	40

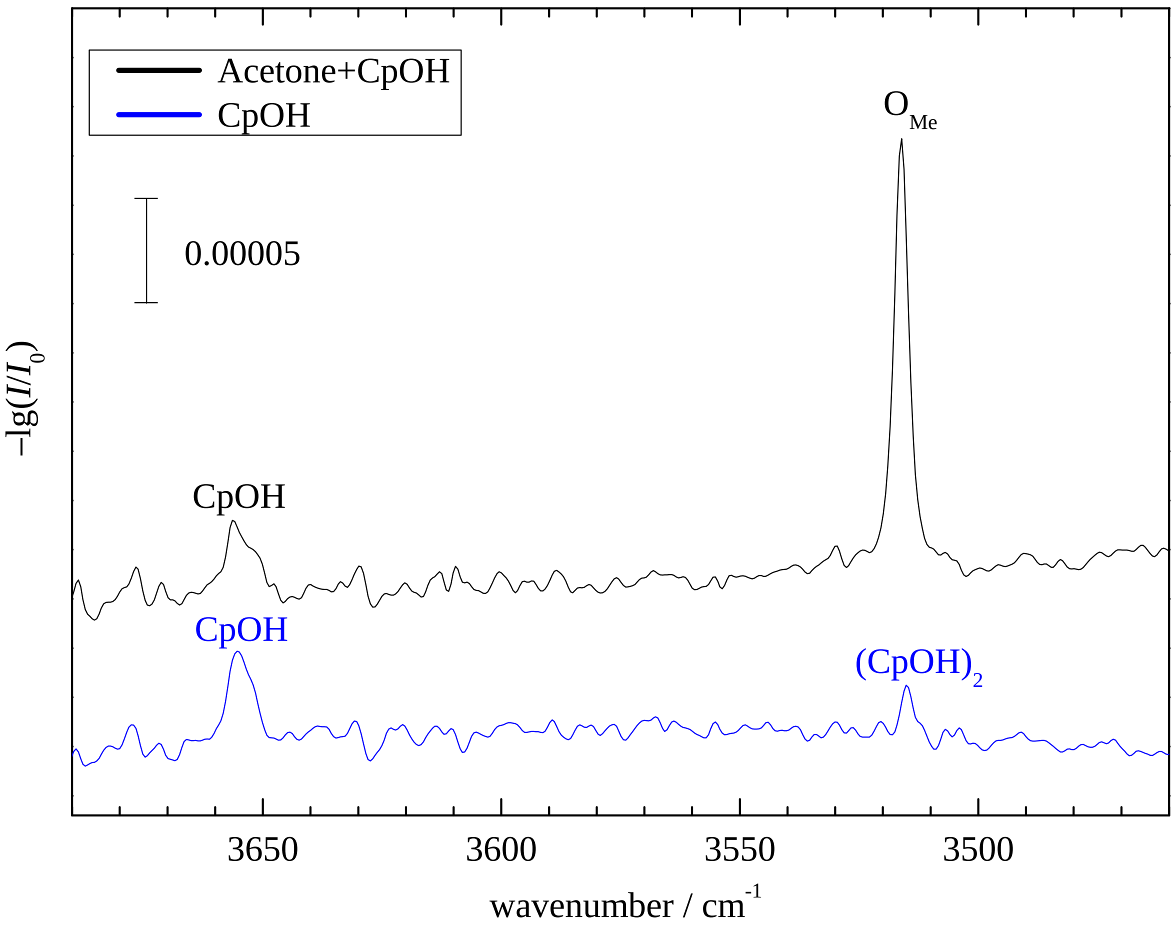

Fig. S6: OH stretching FTIR spectra of CpOH expansions in He with (black) and without acetone (blue) under otherwise identical experimental conditions. The signals of the homodimer $(\mathrm{CpOH})_2$ and of the 1:1 complex $\mathrm{O_{Me}}$ (no docking isomerism because of the symmetry of acetone) are seen to overlap almost perfectly. This overlap is removed for pinacolone, likely because the interaction of the Cp ring with the *t*Bu group on the other side of the ketone group distorts the hydrogen bond.

Table S7: Experimental range for the integrated intensity ratios $I_{\mathrm{Me}}/I_{t\mathrm{Bu}}$, with absorption cross-sections from theory (two different DFT functionals and basis sets), derived docking ratios $c_{\mathrm{Me}}/c_{t\mathrm{Bu}}$ and resulting experimental fractions $x_{t\mathrm{Bu}}$ for *t*Bu docking. The minimal and maximal values for $I_{\mathrm{Me}}/I_{t\mathrm{Bu}}$ where received using an automated statistical evaluation[S4] and were used to determine the span of possible $c_{\mathrm{Me}}/c_{t\mathrm{Bu}}$ and $x_{t\mathrm{Bu}}$ values consistent with the spectra, assuming exact theoretical cross section ratios.

Method	Donor	$\frac{I_{\mathrm{Me}}}{I_{t\mathrm{Bu}}}$	$\frac{c_{\mathrm{Me}}}{c_{t\mathrm{Bu}}}$	$x_{t\mathrm{Bu}}$
B3LYP-D3(BJ)/def2-TZVP	MeOH	5.91-11.82	4.51-9.02	0.10-0.18
	*t*BuOH	2.38-4.16	1.70-2.96	0.25-0.37
	CpOH	5.54-7.30	3.36-4.42	0.18-0.23
B3LYP-D3(BJ)/def2-QZVP	MeOH	5.91-11.82	4.14-8.28	0.11-0.19
	*t*BuOH	2.38-4.16	1.69-2.95	0.25-0.37
	CpOH	5.54-7.30	3.38-4.44	0.18-0.23
TPSS-D3(BJ)/def2-TZVP	MeOH	5.91-11.82	4.03-8.06	0.11-0.20
	*t*BuOH	2.38-4.16	1.53-2.67	0.27-0.40
	CpOH	5.54-7.30	3.46-4.56	0.18-0.22
TPSS-D3(BJ)/def2-QZVP	MeOH	5.91-11.82	4.39-8.77	0.10-0.19
	*t*BuOH	2.38-4.16	1.54-2.68	0.27-0.39
	CpOH	5.54-7.30	3.18-4.18	0.19-0.24

Table S8: Experimental range for the integrated intensity ratios $I_{t\mathrm{Bu}}/I_{\mathrm{Me}}$, with absorption cross-sections from theory (two different DFT functionals and basis sets), derived docking ratios $c_{t\mathrm{Bu}}/c_{\mathrm{Me}}$ and resulting experimental fractions x_{Me} for methyl docking. The minimal and maximal values for $I_{t\mathrm{Bu}}/I_{\mathrm{Me}}$ where obtained using an automated statistical evaluation[S4] and were used to determine the span of possible $c_{t\mathrm{Bu}}/c_{\mathrm{Me}}$ and x_{Me} values consistent with the spectra. Differences to the inverse evaluation in Tab. S7 are small.

Method	Donor	$\frac{I_{t\mathrm{Bu}}}{I_{\mathrm{Me}}}$	$\frac{c_{t\mathrm{Bu}}}{c_{\mathrm{Me}}}$	x_{Me}
B3LYP-D3(BJ)/def2-TZVP	MeOH	0.08-0.17	0.11-0.22	0.82-0.90
	*t*BuOH	0.24-0.42	0.34-0.59	0.63-0.75
	CpOH	0.14-0.18	0.23-0.29	0.77-0.81
B3LYP-D3(BJ)/def2-QZVP	MeOH	0.08-0.17	0.12-0.24	0.81-0.89
	*t*BuOH	0.24-0.42	0.34-0.59	0.63-0.75
	CpOH	0.14-0.18	0.23-0.29	0.78-0.81
TPSS-D3(BJ)/def2-TZVP	MeOH	0.08-0.17	0.12-0.25	0.80-0.89
	*t*BuOH	0.24-0.42	0.37-0.65	0.61-0.73
	CpOH	0.14-0.18	0.23-0.28	0.78-0.82
TPSS-D3(BJ)/def2-QZVP	MeOH	0.08-0.17	0.11-0.23	0.81-0.90
	*t*BuOH	0.24-0.42	0.37-0.65	0.61-0.73
	CpOH	0.14-0.18	0.25-0.31	0.76-0.80

Table S9: Electronic dissociation energies of alcohol-pinacolone complexes for two different DFT functionals and basis sets in $\mathrm{kJ\,mol^{-1}}$. Alcohol and docking variations are of comparable magnitude and the difference between the two docking options never exceeds $8\,\%$.

Donor	Method	Me	*t*Bu
MeOH	B3LYP-D3(BJ)/def2-TZVP	33.4	32.0
	B3LYP-D3(BJ)/def2-QZVP	30.9	29.3
	TPSS-D3(BJ)/def2-TZVP	32.4	30.2
	TPSS-D3(BJ)/def2-QZVP	30.1	27.9
*t*BuOH	B3LYP-D3(BJ)/def2-TZVP	34.1	33.5
	B3LYP-D3(BJ)/def2-QZVP	31.8	31.0
	TPSS-D3(BJ)/def2-TZVP	32.9	31.4
	TPSS-D3(BJ)/def2-QZVP	30.7	29.1
CpOH	B3LYP-D3(BJ)/def2-TZVP	35.9	35.5
	B3LYP-D3(BJ)/def2-QZVP	33.6	33.2
	TPSS-D3(BJ)/def2-TZVP	33.8	32.4
	TPSS-D3(BJ)/def2-QZVP	31.7	30.2

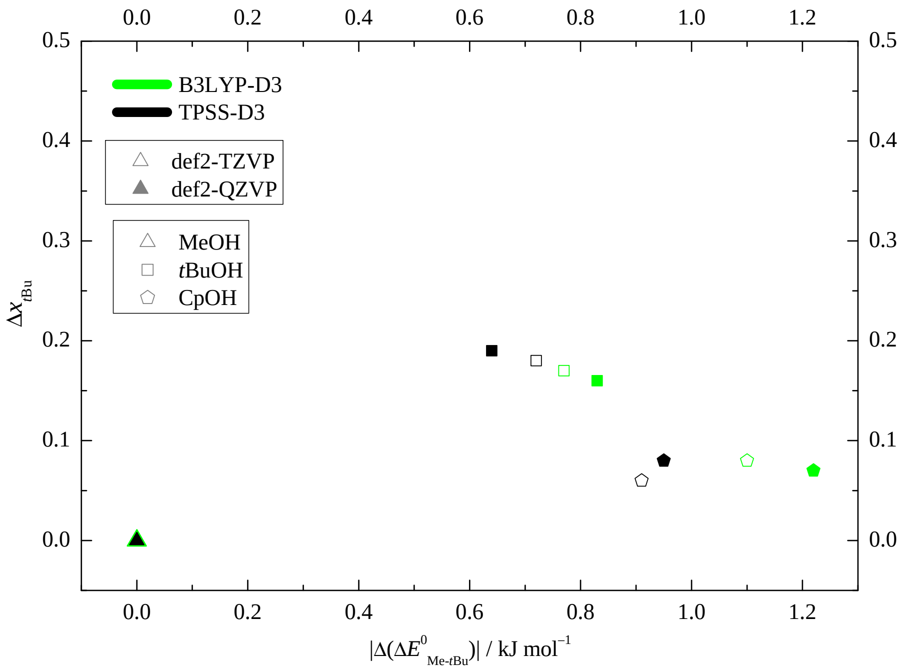

Fig. S7: The experimental *t*Bu docking abundance gain $\Delta x_{t\mathrm{Bu}}$ plotted against the theoretical *t*Bu docking energy gain $\Delta\Delta E^0_{t\mathrm{Bu}}$. It illustrates the alcohol substitution trend from MeOH (△) to *t*BuOH (□) and to CpOH (⬠). Theory predicts more energy gain for CpOH across all methods but experiment shows more abundance gain for *t*BuOH. The London dispersion gain of the *t*Bu side is significant in both cases, but not sufficient to tip the balance towards *t*Bu docking (see also Fig. 6).

Table S10: Effective conformational freezing temperatures T_c for alcohol-pinacolone systems, calculated with ZPVE corrected DFT energy differences (in parentheses based on DLPNO-CCSD(T) electronic energies from Tab. S12 instead of DFT electronic energies) and the band integral ratios obtained from experiment. Error bars were obtained from upper and lower band integral ratio bounds and chosen symmetrically. For the barriers expected in these systems, plausible T_c values are $\leq 150\,\text{K}$.[S5] and positive, but significantly higher than the rotational temperature of about $10\,\text{K}$. A higher T_c indicates an overestimated energy gap (or an unexpectedly high barrier) and a very low T_c indicates an underestimated energy gap. B3LYP is thus seen to favour *t*Bu docking too much for CpOH and TPSS to disfavour *t*Bu somewhat for *t*BuOH. The CCSD(T) correction (in parentheses) switches the docking sequence for CpOH and lowers all *t*BuOH values too much, leaving only MeOH predictions in a plausible range.

Donor	Method	def2-TZVP	def2-QZVP
		T_c/K	T_c/K
	B3LYP-D3(BJ)	94 ± 18	112 ± 22
		(44 ± 8)	(84 ± 16)
MeOH	TPSS-D3(BJ)	147 ± 29	147 ± 28
		(55 ± 11)	(54 ± 10)
	B3LYP-D3(BJ)	105 ± 36	128 ± 44
		(29 ± 10)	(19 ± 6)
*t*BuOH	TPSS-D3(BJ)	268 ± 106	303 ± 118
		(4 ± 2)	(8 ± 3)
	B3LYP-D3(BJ)	26 ± 3	33 ± 3
		(-29 ± 3)	(-20 ± 2)
CpOH	TPSS-D3(BJ)	99 ± 10	113 ± 12
		(-20 ± 2)	(-26 ± 3)

Table S11: LED analysis for the methyl and *tert*-butyl bound B3LYP and TPSS minimum structures at DLPNO-CCSD(T) level (single point computations) in $\mathrm{kJ\,mol^{-1}}$. The interfragment dispersion contributions of strong and weak pairs (ORCA LED output) were combined to yield the total dispersion contribution to the intermolecular interaction. ΔD in the last column gives the difference in dispersion contribution between both docking sides. Its consistently positive value shows that the *tert*-butyl docking structure is offering more dispersion attraction than the corresponding methyl docking structure. CpOH profits most from this attraction, at least at B3LYP level.

pre-optimization level		With D $\Delta E^{\mathrm{el}}_{\mathrm{Me}-t\mathrm{Bu}}$	Without D $\Delta E^{\mathrm{el}}_{\mathrm{Me}-t\mathrm{Bu}}$	ΔD
B3LYP-D3(BJ)/def2-TZVP	MeOH	−0.65	−3.09	+2.44
	*t*BuOH	−0.21	−2.39	+2.19
	CpOH	+0.26	−2.88	+3.14
B3LYP-D3(BJ)/def2-QZVP	MeOH	−1.17	−3.50	+2.32
	*t*BuOH	−0.16	−2.53	+2.37
	CpOH	+0.21	−2.91	+3.13
TPSS-D3(BJ)/def2-TZVP	MeOH	−0.99	−3.12	+2.12
	*t*BuOH	−0.20	−2.28	+2.08
	CpOH	−0.02	−2.30	+2.28
TPSS-D3(BJ)/def2-QZVP	MeOH	−0.85	−2.37	+1.52
	*t*BuOH	−0.19	−2.42	+2.23
	CpOH	0.00	−2.33	+2.33

Table S12: Energy advantage of methyl relative to *t*-butyl coordination of pinacolone by three alcohols at DFT and DLPNO-CCSD(T) corrected level in $\mathrm{kJ\,mol^{-1}}$. ΔE^0 includes and ΔE^{el} excludes the harmonic DFT ZPVE. Higher stability for the *t*-butyl coordination (contradicting experiment) is indicated by a positive energy difference. The CCSD(T) correction leads to a systematic shift towards *t*-butyl docking, in particular for TPSS structures.

Donor	Method	$\Delta E^{\mathrm{el}}_{\mathrm{Me}-t\mathrm{Bu}}$	$\Delta E^{0}_{\mathrm{Me}-t\mathrm{Bu}}$
MeOH	B3LYP-D3(BJ)/def2-TZVP	−1.39	−1.40
	DLPNO-CCSD(T)//B3LYP-D3(BJ)/def2-TZVP	−0.65	−0.66
	B3LYP-D3(BJ)/def2-QZVP	−1.58	−1.59
	DLPNO-CCSD(T)//B3LYP-D3(BJ)/def2-QZVP	−1.17	−1.19
	TPSS-D3(BJ)/def2-TZVP	−2.27	−2.04
	DLPNO-CCSD(T)//TPSS-D3(BJ)/def2-TZVP	−0.99	−0.76
	TPSS-D3(BJ)/def2-QZVP	−2.22	−2.16
	DLPNO-CCSD(T)//TPSS-D3(BJ)/def2-QZVP	−0.85	−0.78
*t*BuOH	B3LYP-D3(BJ)/def2-TZVP	−0.66	−0.62
	DLPNO-CCSD(T)//B3LYP-D3(BJ)/def2-TZVP	−0.21	−0.17
	B3LYP-D3(BJ)/def2-QZVP	−0.80	−0.75
	DLPNO-CCSD(T)//B3LYP-D3(BJ)/def2-QZVP	−0.16	−0.11
	TPSS-D3(BJ)/def2-TZVP	−1.50	−1.32
	DLPNO-CCSD(T)//TPSS-D3(BJ)/def2-TZVP	−0.20	−0.02
	TPSS-D3(BJ)/def2-QZVP	−1.66	−1.51
	DLPNO-CCSD(T)//TPSS-D3(BJ)/def2-QZVP	−0.19	−0.04
CpOH	B3LYP-D3(BJ)/def2-TZVP	−0.35	−0.29
	DLPNO-CCSD(T)//B3LYP-D3(BJ)/def2-TZVP	+0.26	+0.32
	B3LYP-D3(BJ)/def2-QZVP	−0.38	−0.37
	DLPNO-CCSD(T)//B3LYP-D3(BJ)/def2-QZVP	+0.21	+0.23
	TPSS-D3(BJ)/def2-TZVP	−1.37	−1.12
	DLPNO-CCSD(T)//TPSS-D3(BJ)/def2-TZVP	−0.02	+0.23
	TPSS-D3(BJ)/def2-QZVP	−1.48	−1.21
	DLPNO-CCSD(T)//TPSS-D3(BJ)/def2-QZVP	0.00	+0.28

Table S13: D3 contribution analysis in $\mathrm{kJ\,mol^{-1}}$ to the energy advantage of the methyl side at the D3-inclusive optimized structures after zero point energy correction for the B3LYP and TPSS functionals. ΔD3 in the last column gives the dispersion correction advantage for *tert*-butyl docking. A positive value means that the *tert*-butyl docking structure offers more D3 attraction than the corresponding methyl docking structure. The effects are quite similar to those of the LED analysis in Tab. S11.

		With D3		Without D3		
	Donor	$\Delta E^{\mathrm{el}}_{\mathrm{Me}-t\mathrm{Bu}}$	$\Delta E^{0}_{\mathrm{Me}-t\mathrm{Bu}}$	$\Delta E^{\mathrm{el}}_{\mathrm{Me}-t\mathrm{Bu}}$	$\Delta E^{0}_{\mathrm{Me}-t\mathrm{Bu}}$	ΔD3
	MeOH	−1.39	−1.40	−3.44	−3.44	+2.05
B3LYP-D3(BJ)	*t*BuOH	−0.66	−0.62	−2.79	−2.76	+2.14
/def2-TZVP	CpOH	−0.35	−0.29	−3.13	−3.07	+2.78
	MeOH	−1.58	−1.59	−3.94	−3.95	+2.37
B3LYP-D3(BJ)	*t*BuOH	−0.80	−0.75	−2.43	−2.38	+1.63
/def2-QZVP	CpOH	−0.38	−0.37	−3.17	−3.15	+2.79
	MeOH	−2.27	−2.04	−4.24	−4.02	+1.98
TPSS-D3(BJ)	*t*BuOH	−1.50	−1.32	−3.12	−2.94	+1.62
/def2-TZVP	CpOH	−1.37	−1.12	−3.07	−2.82	+1.70
	MeOH	−2.22	−2.16	−3.75	−3.69	+1.53
TPSS-D3(BJ)	*t*BuOH	−1.66	−1.51	−3.27	−3.13	+1.61
/def2-QZVP	CpOH	−1.48	−1.21	−3.31	−3.03	+1.83

Table S14: Exploration of alternative CpOH docking structures (Me1, *t*Bu1). Docking energy advantages in $\mathrm{kJ\,mol^{-1}}$ without (ΔE^{el}) and with (ΔE^{0}) zero point energy correction and corresponding wavenumber shifts $\Delta\omega$ in $\mathrm{cm^{-1}}$ predicted by four DFT levels. Positive energy values indicate a higher stability of the second docking variant listed under Comparison. Me1-*t*Bu1 appears to fit experiment (Tab. S6) better than Me-*t*Bu in relative energy and shifts. However, DLPNO-CCSD(T) level calculations (see Tab. S15) discourage this alternative.

		def2-TZVP			def2-QZVP		
Comparison	Method	ΔE^{el}	ΔE^{0}	$\Delta\omega$	ΔE^{el}	ΔE^{0}	$\Delta\omega$
Me-*t*Bu	B3LYP-D3(BJ)	-0.35	-0.29	-51	-0.38	-0.37	-51
	TPSS-D3(BJ)	-1.37	-1.12	-50	-1.48	-1.21	-63
Me1-*t*Bu1	B3LYP-D3(BJ)	-1.03	-0.98	-38	-0.94	-0.93	-40
	TPSS-D3(BJ)	-1.50	-1.23	-43	-1.36	-1.36	-36
Me-Me1	B3LYP-D3(BJ)	-1.38	-1.07	$+23$	-1.28	-1.02	$+25$
	TPSS-D3(BJ)	-1.18	-1.00	$+23$	-1.02	-0.89	$+22$
tBu-*t*Bu1	B3LYP-D3(BJ)	-2.05	-1.76	$+35$	-1.84	-1.59	$+36$
	TPSS-D3(BJ)	-1.31	-1.10	$+31$	-0.90	-1.05	$+48$
Me1-*t*Bu	B3LYP-D3(BJ)	$+1.02$	$+0.77$	-75	$+0.90$	$+0.65$	-75
	TPSS-D3(BJ)	-0.19	-0.12	-74	-0.46	-0.32	-85
Me-*t*Bu1	B3LYP-D3(BJ)	-2.40	-2.05	-15	-2.22	-1.95	-15
	TPSS-D3(BJ)	-2.68	-2.23	-19	-2.38	-2.25	-15

Table S15: Ruling out alternative CpOH docking structures (Me1, *t*Bu1) explored in Tab. S14 based on DLPNO-CCSD(T) single point calculations at the structures obtained by different preoptimization (Preopt.) methods. The energy penalty of both alternatives (in $\mathrm{kJ\,mol^{-1}}$) further increases. Also given is the difference in dispersion contribution $\Delta\mathrm{D}$ between the docking sides as in Tab. S11. Positive values indicate more dispersion interaction in the second docking variant listed than in the first. Me1 and *t*Bu1 are thus less dispersively bound.

		def2-TZVP		def2-QZVP	
Comparison	Preopt.	$\Delta E^{\mathrm{el+D}}$	$\Delta\mathrm{D}$	$\Delta E^{\mathrm{el+D}}$	$\Delta\mathrm{D}$
Me-*t*Bu	B3LYP-D3(BJ)	$+0.26$	$+3.14$	$+0.21$	$+3.13$
	TPSS-D3(BJ)	-0.02	$+2.28$	0.00	$+2.33$
Me1-*t*Bu1	B3LYP-D3(BJ)	-0.26	$+2.71$	-0.30	$+2.30$
	TPSS-D3(BJ)	-0.27	$+2.85$	-0.57	$+2.16$
Me-Me1	B3LYP-D3(BJ)	-1.93	-4.93	-1.93	-4.95
	TPSS-D3(BJ)	-1.93	-4.54	-1.92	-4.14
tBu-*t*Bu1	B3LYP-D3(BJ)	-2.44	-5.36	-2.44	-5.79
	TPSS-D3(BJ)	-2.18	-3.97	-2.49	-4.31
Me1-*t*Bu	B3LYP-D3(BJ)	$+2.20$	$+8.06$	$+2.15$	$+8.08$
	TPSS-D3(BJ)	$+1.92$	$+6.82$	$+1.92$	$+6.47$
Me-*t*Bu1	B3LYP-D3(BJ)	-2.20	-2.22	-2.23	-2.66
	TPSS-D3(BJ)	-2.20	-1.69	-2.49	-1.98

Table S16: Cartesian coordinates for MeOH-pinacolone computed at B3LYP-D3(BJ,abc)/def2-QZVP level, with an electronic energy of $\Delta E_{\mathrm{el}} = -426.802\,316\,05\,E_{\mathrm{h}}$. Calculations were carried out with ORCA 4.2.1.[S1]

	Me sided structure		
Atom	X	Y	Z
C	-1.50750299522325	0.12174811338038	-0.95517936921608
C	-2.57075086631565	-0.17467193411340	0.10440548509532
H	-2.36421770897261	-1.11003976094664	0.61915782365618
H	-2.60703931414104	0.61350424821523	0.85527675627404
H	-3.55058957694961	-0.24543530635660	-0.36799061538118
C	-1.47117953104608	-1.02770533087187	-1.98347683490800
H	-0.74491173462978	-0.84692345029944	-2.77462174352207
H	-1.22770590262443	-1.97480173022255	-1.50258081252406
H	-2.45251695974972	-1.12676592813687	-2.44790140399578
C	-1.83254108286195	1.44425077141769	-1.66861893276040
H	-1.82503762881165	2.28191273961513	-0.97053173139976
H	-1.13044459190987	1.66224863208578	-2.47149166006445
H	-2.82887790950832	1.38491365718235	-2.10665639903773
C	-0.12289141340772	0.19438488374202	-0.30176688251005
O	0.04977115530390	-0.19978963045347	0.83475929587004
C	1.02662722090502	0.76460344736385	-1.09855058058763
H	1.97157366033013	0.43026333794603	-0.67961879799976
H	0.96485291843296	0.50789776365985	-2.15370410049169
H	0.98712625786161	1.85389176487643	-1.02914984159978
C	3.10413168852774	-1.19028934604371	2.58434617341549
H	4.18573443767188	-1.17226041888578	2.71028444611265
H	2.64295605213489	-0.99351247907875	3.55788055487623
H	2.81369373127507	-2.19580773330141	2.26125609403936
O	2.76787542486281	-0.20417472284116	1.62698176696966

H	1.80815917829057	-0.21527758011408	1.48589618507400

Table S17: Cartesian coordinates for MeOH-pinacolone computed at B3LYP-D3(BJ,abc)/def2-QZVP level, with an electronic energy of $\Delta E_{\mathrm{el}} = -426.801\,715\,34\,E_{\mathrm{h}}$. Calculations were carried out with ORCA 4.2.1.[S1]

	*t*Bu sided structure		
Atom	X	Y	Z
C	-0.97363749210914	-0.38241403702950	-0.81599995679926
C	-0.18797526756486	-1.69833434329797	-0.85982903972026
H	0.85457289266806	-1.55651238059902	-0.59173023579138
H	-0.61885321096436	-2.43405319672184	-0.18031852031488
H	-0.22527218449034	-2.10756912566208	-1.86953874428409
C	-0.29286135932791	0.65689915895111	-1.73429646810484
H	-0.81903674330938	1.61218201486691	-1.72171277087247
H	0.74090472969075	0.81982640418979	-1.43398790517317
H	-0.29900504846198	0.28695440939468	-2.76002921051303
C	-2.41326175107012	-0.62716812034222	-1.28935571924913
H	-2.93631912440231	-1.32537512942836	-0.63471254522172
H	-2.99451902779030	0.29183156963411	-1.34367389053172
H	-2.39455763303589	-1.06262040930693	-2.28817822565549
C	-0.92973603289032	0.21361560306079	0.59665305989849
O	0.01290004372825	0.01721193089620	1.33731996299323
C	-2.06334040200076	1.10258135380549	1.04908841994165
H	-2.96536232367447	0.50740722261174	1.19863000461824
H	-1.79240267715170	1.58636555490301	1.98257422127218
H	-2.30119668876034	1.85193514415329	0.29438104211797
C	3.55682720781985	0.66063899663263	1.18879806502573
H	4.50294977565727	0.76940182169037	0.66077807934431
H	3.20368268290009	1.65813821111530	1.47056030683340
H	3.73795763848203	0.08627908538382	2.10302794660443

O	2.65519172325491	0.00325890383357	0.31743988575601
H	1.80031985668638	-0.08839603709070	0.76280760365080

Table S18: Cartesian coordinates for *t*BuOH-pinacolone computed at B3LYP-D3(BJ,abc)/def2-QZVP level, with an electronic energy of $\Delta E_{\mathrm{el}} = -544.737\,879\,76\,E_{\mathrm{h}}$. Calculations were carried out with ORCA 4.2.1.[S1]

	Me sided structure		
Atom	X	Y	Z
C	-2.22356265828015	0.79288024100917	1.29323762461125
C	-2.34607924696290	-0.24309740734743	2.41250858093178
H	-2.01784871437348	-1.22471065696712	2.07912543462827
H	-1.73650661435851	0.03348240495112	3.27180244931026
H	-3.38464393538025	-0.31379578892807	2.73601731999016
C	-3.07468036397376	0.34880875354050	0.08531339881449
H	-3.03740560212676	1.07023426809902	-0.72983314624216
H	-2.73985162229509	-0.61611275200831	-0.29468650908594
H	-4.11516128417213	0.24781430018350	0.39459491837931
C	-2.70620018207703	2.16400197284618	1.79256405380991
H	-2.09390388112517	2.52000895810837	2.62168597485943
H	-2.68993498351082	2.91829122309243	1.00776048961103
H	-3.73265111292910	2.07978342776656	2.14947874932826
C	-0.77104584008608	0.87518861685456	0.81215972374542
O	0.03501542980689	0.01884565590993	1.11768341979268
C	-0.36018897603337	2.03649509886853	-0.06136028631022
H	-0.14063711360642	2.89085815516914	0.58274752184973
H	0.53961760153090	1.78181321062118	-0.61426998557793
H	-1.15197243270289	2.34267180159373	-0.74141787677336
C	2.37697912788198	-2.44283387456008	-0.57874128686974
H	2.32953569520490	-3.34213176840628	-1.19444632799063
H	3.33245563336044	-2.43010321390210	-0.05541899737142

C	2.22761505546908	-1.18013049576729	-1.43086992563824
H	1.58026130292315	-2.49705927112411	0.16442017817942
C	3.36923742146342	-1.06653700595703	-2.43448601561576
H	4.32510131549541	-1.03671457863544	-1.91273127839801
H	3.37140818511000	-1.91479620061482	-3.11897653152277
H	3.26972019610989	-0.15037990527941	-3.01618195595549
C	0.87516152610497	-1.17951541834543	-2.15192344785515
H	0.75754508782420	-0.26089191738541	-2.72666483414631
H	0.79128548208534	-2.02660960582057	-2.83396965747354
H	0.05915527506903	-1.24404517286345	-1.43148617400945
O	2.33056551420666	-0.02103650842980	-0.59603942176134
H	1.64707761541716	-0.07422634536062	0.09033713040724

Table S19: Cartesian coordinates for *t*BuOH-pinacolone computed at B3LYP-D3(BJ,abc)/def2-QZVP level, with an electronic energy of $\Delta E_{\mathrm{el}} = -544.737\,575\,86\,E_{\mathrm{h}}$. Calculations were carried out with ORCA 4.2.1.[S1]

	*t*Bu sided structure		
Atom	X	Y	Z
C	-1.65576153024459	-1.46659636984697	0.53253727107518
C	-2.90513307430766	-2.10482905153113	1.15774331258167
H	-3.74521423904310	-1.41392507190191	1.20463575347212
H	-3.21486538887110	-2.95742742366586	0.55383410283170
H	-2.70454514220485	-2.46915028898921	2.16608836388303
C	-0.54726985568465	-2.51758124768499	0.40899708618064
H	-0.23765687317303	-2.87746579126990	1.39009949459593
H	-0.91773690618766	-3.36792357471150	-0.16375703189461
H	0.32760605073904	-2.12444238628488	-0.10044662185761
C	-1.99746169622763	-0.91107330031912	-0.86797306761543
H	-1.11528815025994	-0.47748037594263	-1.33579400306290
H	-2.35563313620917	-1.72608214371422	-1.49761739428758

H	-2.78222414767411	-0.15533376662907	-0.82409425185302
C	-1.16543172171352	-0.27224630638877	1.35851858999109
O	0.01621009042159	-0.00901885506740	1.45549202436433
C	-2.18768201713756	0.61581560556171	2.02830699444557
H	-3.01595467898910	0.84666258376381	1.35980839019625
H	-2.61045975500451	0.10080521081601	2.89251132964629
H	-1.70758731308868	1.53137173526883	2.36038118787636
C	0.90638085616500	2.33758834123403	-1.06998387744679
H	1.27321918380394	3.34773591072016	-1.25646780956324
H	0.11957904458271	2.11718665888567	-1.79136866269626
C	2.03464901958627	1.30853380727355	-1.19060912419190
H	0.47468352379383	2.31177432038632	-0.06902989239490
C	2.61395463483609	1.30664344908272	-2.60020865868316
H	3.39417095041274	0.55101483062029	-2.68480616382701
H	1.83546004130622	1.07650520319645	-3.32719356328675
H	3.04168474896692	2.27876391236552	-2.84486166151141
C	3.12518996423015	1.58621014074602	-0.15377002399098
H	3.91358851985221	0.83802521664034	-0.22939345382158
H	3.56682007650113	2.57264962495312	-0.30143146828145
H	2.71027180917342	1.54732414598379	0.85430005599617
O	1.50576528239252	-0.00895201546982	-0.99000979492100
H	1.15260961807506	-0.06429213302201	-0.09026296659674

Table S20: Cartesian coordinates for CpOH-pinacolone computed at B3LYP-D3(BJ,abc)/def2-QZVP level, with an electronic energy of $\Delta E_{\text{el}} = -582.828\,647\,41\,E_{\text{h}}$. Calculations were carried out with ORCA 4.2.1.[S1]

	Me sided structure		
Atom	X	Y	Z
C	-2.27748468514331	-0.62127761315438	1.28455507614650
C	-2.80443921143852	-0.19788573051747	-0.10209294821137

H	-2.32644724973454	0.72164639848871	-0.43860422274116
H	-2.63723763505870	-0.96507313683172	-0.85670014575362
H	-3.87802050163541	-0.01863247780382	-0.04017956876471
C	-2.57475183484921	0.48180335142541	2.30190712004360
H	-2.19485693765751	0.21957012852582	3.28839276911644
H	-2.11150462635781	1.42177705755180	2.01092909386309
H	-3.65176380677728	0.63288856187281	2.37640414156008
C	-2.95223895003493	-1.93211993318822	1.72023105431164
H	-2.80834518514810	-2.73106426928087	0.99494615602116
H	-2.56930577866317	-2.27160547487914	2.68318353727501
H	-4.02497610082364	-1.77045619766978	1.82671894834514
C	-0.76442415866917	-0.82072242771659	1.15410693345337
O	0.01384260702680	-0.02420240031184	1.64007024124392
C	-0.25081118938653	-2.02593741914161	0.40174273304047
H	-0.93068509354675	-2.35611830217873	-0.37942469206589
H	0.72838166376673	-1.80429522053112	-0.01462266658995
H	-0.14438930799171	-2.84937894085231	1.11177243997035
C	3.06998074846097	0.89428131618468	-2.03815878125634
C	1.86364575382356	0.34742004133883	-2.83056471320268
C	0.60372842323083	0.95128376288962	-2.14984062307762
C	1.13440933541488	1.82344284794360	-0.99432725734815
C	2.50426404584876	1.22572446541995	-0.66070681331615
O	2.39518288273706	0.00152383606548	0.06167791723728
H	3.44955593321727	1.80949950481957	-2.49626805725558
H	3.89481575823666	0.18732296052898	-1.97008016203915
H	1.83837142909631	-0.73920930487751	-2.77427381272607
H	1.92419321039128	0.61118865958672	-3.88550460293024
H	-0.03404430251363	0.15691419652282	-1.76574624978906
H	-0.00154924738952	1.53182078519604	-2.84484865970948

H	0.47199440421480	1.84989402390182	-0.13031270146819
H	1.27769898589565	2.85357676228835	-1.32755513236316
H	3.13640566994248	1.92864449794015	-0.10860749111314
H	1.73992087093339	0.09852573002064	0.76827565835966

Table S21: Cartesian coordinates for CpOH-pinacolone computed at B3LYP-D3(BJ,abc)/def2-QZVP level, with an electronic energy of $\Delta E_{\mathrm{el}} = -582.828\,502\,85\,E_{\mathrm{h}}$. Calculations were carried out with ORCA 4.2.1.[S1]

	*t*Bu sided structure		
Atom	X	Y	Z
C	-1.40571637591276	-2.12956340681337	0.29594056506736
C	-0.15286617958567	-2.34467974985408	1.16890373007603
C	-0.29639102440529	-1.32916147572880	2.30500292437228
C	-1.78871959690498	-1.37533439701115	2.61628848834612
C	-2.47009293560537	-1.50048511601163	1.23694006912625
H	-1.74934566089945	-3.06290213553468	-0.14780071159976
H	0.78128043326057	-2.22292716920049	0.62212759966241
H	-0.15293750079859	-3.34859381541796	1.59876700509210
H	-2.76463256850693	-0.51848314486125	0.87109689669079
H	-3.37528011927234	-2.10358127974405	1.29118477680223
H	-1.18348953383821	-1.45267352656178	-0.52725064730151
H	-2.10641049596081	-0.49638643609362	3.17434899445679
H	-1.99567233531261	-2.25412725624366	3.22963911053165
H	0.31914437113007	-1.58906518286979	3.17180576758206
O	0.00921245438293	-0.00181629387740	1.87680714836697
H	0.82137610971685	-0.01343263291701	1.35382082158800
C	0.59811862063279	1.79690971258064	-1.33006538851002
C	1.13907068820179	2.66505087253493	-0.19062010490052
H	0.92239039865896	2.22935390139596	0.78080103969987
H	2.21800015423665	2.79358039574891	-0.27192687176879

H	0.67301479696503	3.64967772525001	-0.23230632098102
C	-0.91550910198059	1.57006934635914	-1.12533470349689
H	-1.34891235117333	0.96476207830616	-1.92126974256479
H	-1.10436371106903	1.08453009786969	-0.16971907400030
H	-1.42345851896577	2.53498190650778	-1.12807067797989
C	0.84350993518386	2.49467048730055	-2.67750949363211
H	1.90912329641162	2.63088300206825	-2.86662076226194
H	0.41556540887246	1.94312313938325	-3.51300793308630
H	0.38062095905472	3.48120380935039	-2.66269324313515
C	1.26622000997373	0.41821626625616	-1.30808786204082
O	1.85380530719137	0.00864429540664	-0.32760904849475
C	1.16638859636088	-0.45357809450876	-2.53905093765105
H	1.39913861636577	-1.48016822827861	-2.27219111058073
H	0.18273140142587	-0.39852556185924	-3.00132877987240
H	1.89001276396647	-0.11080049044062	-3.28103055909100

References

(S1) Neese, F. Software update: the ORCA program system, version 4.0. *WIREs Comput. Mol. Sci.* **2018**, *8*, e1327.

(S2) TURBOMOLE V7.3 2018, a development of University of Karlsruhe and Forschungszentrum Karlsruhe GmbH, 1989-2007, TURBOMOLE GmbH, since 2007; available from `http://www.turbomole.com`.

(S3) Furche, F.; Ahlrichs, R.; Hättig, C.; Klopper, W.; Sierka, M.; Weigend, F. Turbomole. *WIREs Comput. Mol. Sci.* **2014**, *4*, 91–100.

(S4) Karir, G.; Lüttschwager, N. O. B.; Suhm, M. A. Phenylacetylene as a gas phase sliding balance for solvating alcohols. *Phys. Chem. Chem. Phys.* **2019**, *21*, 7831–7840.

(S5) Poblotzki, A.; Gottschalk, H. C.; Suhm, M. A. Tipping the Scales: Spectroscopic Tools for Intermolecular Energy Balances. *The Journal of Physical Chemistry Letters* **2017**, *8*, 5656–5665.
